# Cell‐Size Confinement Drives Size‐Dependent Scaling of Intracellular Reaction‐Diffusion Waves for Robust Patterning

**DOI:** 10.1002/smsc.202500643

**Published:** 2026-04-23

**Authors:** Sakura Takada, Shunshi Kohyama, Miho Yanagisawa, Nobuhide Doi, Natsuhiko Yoshinaga, Kei Fujiwara

**Affiliations:** ^1^ Department of Biosciences and Informatics Faculty of Science and Technology Keio University Yokohama Kanagawa Japan; ^2^ Komaba Institute for Science Graduate School of Arts and Sciences The University of Tokyo Meguro‐ku Tokyo Japan; ^3^ Graduate School of Science The University of Tokyo Meguro‐ku Tokyo Japan; ^4^ Center for Complex Systems Biology Universal Biology Institute The University of Tokyo Meguro‐ku Tokyo Japan; ^5^ Department of Complex and Intelligent systems Future University Hakodate Hakodate Hokkaido Japan

**Keywords:** artificial cells, cell division, cell‐size space effect, reaction‐diffusion system, scaling, spatiotemporal regulation, synthetic biology

## Abstract

Intracellular reaction–diffusion (iRD) waves are a mechanism to set the positions of molecules within cells for their proper biological function. Despite their biological importance, the physical characteristics of iRD waves were still elusive due to the specific constraints of cells, where spatial confinement and surface‐bulk coupling govern pattern formation. Here, by an artificial cell experiment of iRD wave for cell division (Min wave) using defined factors, we showed that the wavelength of iRD wave is selected by the space size. This wavelength selection adjusted the wave shape and speed to fit the spatial sizes. Furthermore, this mechanism conferred the robustness of the macroscopic pattern of iRD waves under physicochemical perturbations. Theoretical analysis confirmed that this can be explained by the theoretical Min wave model and suggests its generality among iRD waves. These findings indicate the reason why cells use RD waves to place molecules with versatile functions.

## Graphical abstract

Intracellular reaction‐diffusion (iRD) waves scale their wavelength to space size when they are confined within cell‐size spaces, despite having intrinsic wavelength in open systems. This wavelength selection ensures the scaling of the wave shape and the velocity, preserving these essential properties against physicochemical perturbations. This scaling mechanism explains how cells employ iRD waves to achieve precise molecular positioning.

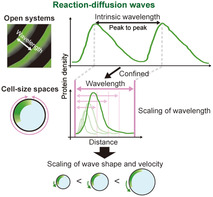

## 1 Introduction

The spatial organization of molecules within living cells underpins essential biological processes ranging from intracellular homeostasis to directed cell migration. Recent studies have revealed that such molecular placements are not merely determined by the self‐assembly of molecules, such as liquid–liquid phase separation, but emerge from self‐organization as a course of molecular reaction networks [1, 2, 3]. A central mechanism underlying the dynamic organization of molecules in living cells is the reaction‐diffusion (RD) system, which shape patterns arising from the coupling of chemical reactions and molecular diffusion under far‐from‐equilibrium conditions [4, 5, 6]. Although RD systems are famous for shaping the spatiotemporal patterns of life, such as animal skin patterns [7] and fingerprints [8], recent studies have shown that they also function as a mechanism for shaping intracellular structures. These patterns formed by RD system have been shown to regulate various cellular activities such as polarity [9, 10], migration [11, 12], and division site selection [13].

In cells, the pattern formation mechanism by RD system has been classified into several groups. The most famous one is Turing pattern, a stationary pattern under far‐from‐equilibrium conditions. The characteristics of Turing patterns have been widely discussed especially in theoretical contexts, including the case in cell‐like confined geometry and with surface‐bulk coupling [14, 15, 16]. Another famous one is intracellular RD (iRD) waves, which show dynamic movement of the pattern, such as traveling and standing waves. iRD wave is spatially periodic patterns with oscillation in time [4, 17, 18] and is distinct from the traveling front for the boundary of phase separation domains. Previous studies have shown that iRD waves are widely used to determine the position inside cells [13, 18]. iRD waves emerge in confined, cytosol‐membrane (bulk‐surface) coupled systems, where spatial boundaries fundamentally alter wave behaviors compared to bulk systems [19, 20, 21]. To elucidate the characteristics of iRD waves, combining theoretical analysis with the experimental system using defined factors entrapped in cell‐size spaces are necessary. However, there has been a lack of a controlled, quantitative experimental system to analyze how the characteristics of these dynamic waves, such as wave shape and velocity, respond to physical and chemical perturbations, including changes in spatial size and reaction parameters.

So far, the iRD wave for bacterial cell division plane determination (Min waves) [22] is the only iRD wave that can be generated using purified elements in cell‐size spaces [19, 23]. Previous studies have shown that wave formation conditions differ between open and cell‐size systems owing to effects such as mass conservation and lipid interfaces [19, 24]. Min waves also exhibit diverse patterns, from single to multiple waves, and modes, such as traveling and standing waves [19, 25, 26, 27]. Furthermore, the phenomena observed in the Min wave experiments match well with the theoretical model described by the reaction‐diffusion equations [19, 26]. Therefore, the Min wave is the most suitable material for analyzing the characteristics of iRD waves.

In this study, we investigated how cell‐size confinement affects the properties of the Min wave by using an artificial cell system that allows precise control of factor concentrations. Consequently, both experiments and theoretical analysis revealed that the Min wave scales its wavelength with the size of confinement, and it was found that this scaling stabilize positional information of iRD wave under various physicochemical conditions. Furthermore, a reduced theoretical model implicated the generality of the scaling behavior among iRD waves. These findings show that iRD systems inherently possess a self‐regulating mechanism enabling precise and stable spatiotemporal control despite perturbations in closed, cell‐size environments.

## 2 Results and Discussion

**FIGURE 1:**
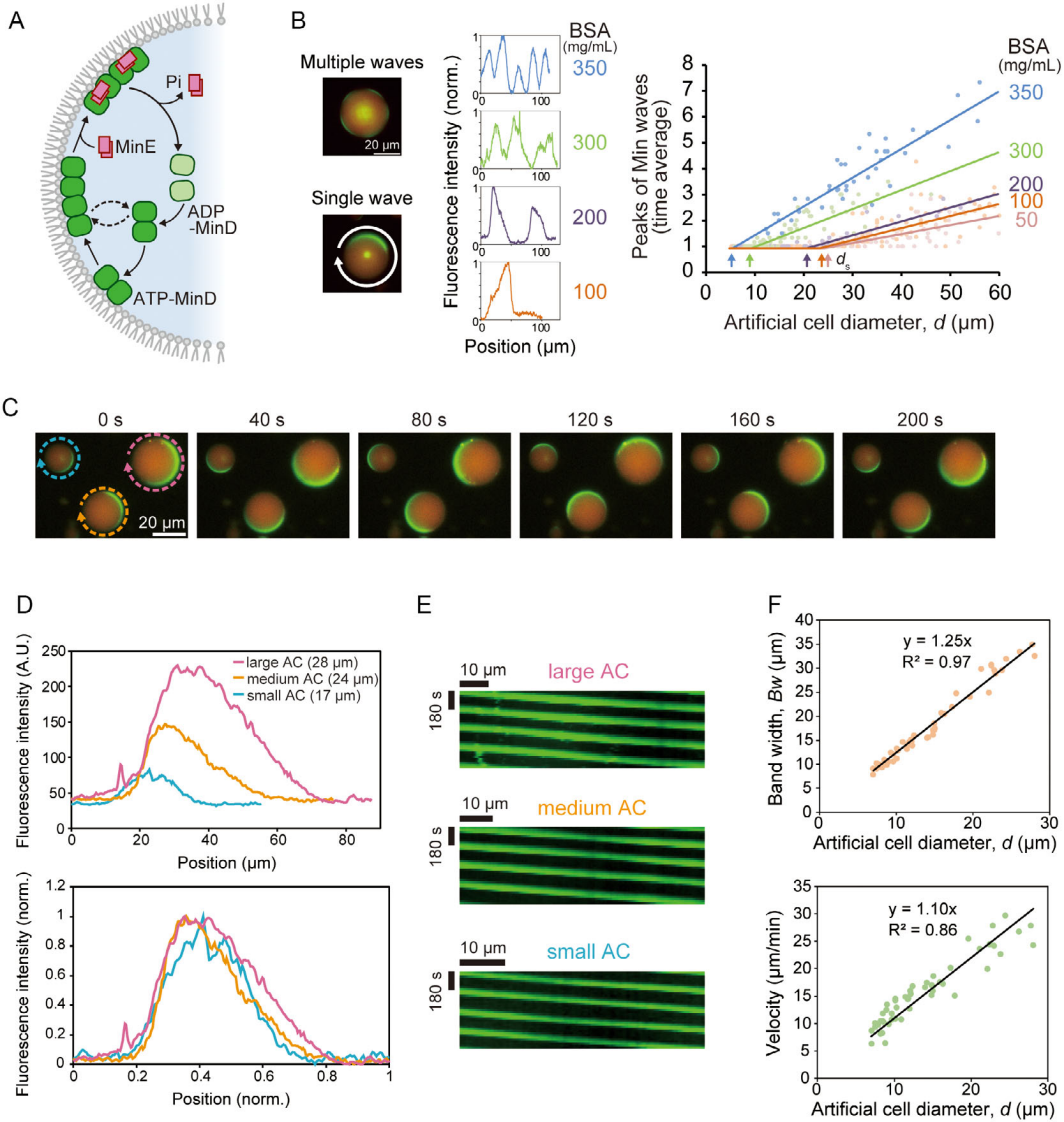
The bandwidth and velocity of a single Min wave scale with the spatial size of the artificial cell. (A) Schematic illustration of the molecular mechanism underlying Min wave generation. (B) Fluorescence images of Min waves reconstituted in microdroplets encapsulating 1 µM sfGFP‐MinD and 1 µM MinE‐mCherry at various BSA concentrations (left). Representative fluorescence images of multiple and single Min waves are shown (left). Fluorescence intensity profiles of sfGFP‐MinD on the membrane (along the white arrow in the left image) of ACs under each BSA concentration are shown (middle). The time‐averaged wave number of Min waves under varying BSA concentrations was analyzed in individual artificial cells and plotted against artificial cell diameters *d* (50 mg/mL BSA; *n* = 100, 100 mg/mL; *n* = 222, 200 mg/mL; *n* = 73, 300 mg/mL; *n* = 73, 350 mg/mL; *n* = 30) (right). Transition points from multiple to single Min waves (*d*_s_) are indicated with arrows. (C–F) Min waves were reconstituted in microdroplets encapsulating 1 µM sfGFP‐MinD, 1 µM MinE‐mCherry, and 100 mg/mL BSA. (C) Time‐lapse images of Min waves in three ACs of different sizes. (D) Fluorescence intensity profiles of sfGFP‐MinD on the membranes of ACs indicated by colored arrows in panel C (top). Intensities and distances are normalized by the maximum and minimum values along each profile and by the circumference length of each artificial cell (bottom). (E) Kymographs of sfGFP‐MinD around the membranes of ACs of varying sizes, corresponding to the colored arrows in panel C. (F) Scatter plots of Min wave bandwidth (top) and velocity (bottom) against artificial cell diameter (*n* = 53). Linear regression lines with zero intercepts are shown.

Min waves can be generated by purified components (MinD, MinE, ATP, and lipids) in bulk [28] and in cell‐size confinement [19, 23]. The mechanism of Min wave generation is based on the coupling of ATP‐dependent membrane binding, protein interactions, and differential diffusion of molecules between lipid membranes and the cytosol [22] (Figure 1A). Since an inhibitor of cell division initiation (MinC) co‐localizes with MinD, Min waves can determine the division plane. Similar to our previous studies [19, 25, 26, 27], water‐in‐oil droplets covered with lipids (polar lipids of *E. coli*) were used as cell‐size spaces. Hereafter, we refer to this cell‐size space as an artificial cell (AC) for simplicity. Proteins fused with fluorescent proteins (sfGFP‐MinD and MinE‐mCherry) and ATP were entrapped in ACs containing 100 mg/mL BSA to simulate living cell conditions. Several minutes after encapsulation, Min waves were observed in almost all artificial cells. Once the waves are generated, they are traveling persistently under our experimental measurements for more than 10 h, indicating that traveling wave of Min wave is not a transient state. Theoretical analysis has shown that Min waves are generated by wave instability [19]. Our focus in this study is not on stationary patterns but on spatiotemporally periodic waves like traveling waves, as they are the main type of pattern observed in Min systems reconstituted in ACs [19, 26]. The ACs in our experimental system do not exhibit heterogeneity, such as a static spatial gradient in membrane physicochemical properties [29] or dynamic front propagation between competent and noncompetent regions [30]. These static or dynamic heterogeneities may induce motion of static wave patterns [29, 30]. As Min waves do not exhibit such heterogeneity, we focus on traveling waves, propagating spatially inhomogeneous Min concentration, under spatially uniform parameters. Although there are theories for generating wave‐like behaviors other than the RD system, both experimental and theoretical studies so far have supported that Min waves are a bona fide iRD wave [19, 21, 26, 28, 31, 32, 33].

First, Min waves in ACs of various sizes were tracked. The results showed that the number of Min wave peaks in the ACs varied with space size (the diameter of the ACs was used as an indicator of space size and is denoted as *d*). In ACs with *d* = 25–250 µm, Min waves show multiple peaks (Figure 1B, Movie S1) as similar to those on lipid planar membranes (in bulk) [28] and in filamentous living cells [34]. Scatter plotting indicates that the peak numbers decrease in smaller ACs, and the degree of decrease has a linear relationship with *d*. In ACs with *d* < 25 µm, the wave peak becomes one (Movie S2), indicating that a single Min wave is generated in ACs. The sizes did not change the fraction of the Min wave modes (Figure S1), and Min waves appeared in ACs with 5 µm diameter or more (Figure 1C). It should be noted that 5 µm diameter is not the lower limit of the spatial size at which the waves appear, but the resolution limit of the microscope setup we used. By increasing the resolution, we have observed Min waves in ACs even with *d* = 2 μm. Therefore, the results hereafter were obtained in spaces that are sufficiently large relative to the critical size d*, a minimal size for Min wave generation. Interestingly, single Min waves are stable and do not exhibit temporal disappearance, even in smaller ACs (*d* ~ 5 µm). Their stability across a range of spatial dimensions (*d* = 5–25 µm) is a characteristic feature specific to single Min waves, as Min waves would otherwise appear stochastically if a linear decrease in the number of peaks were applied to small ACs.

Next, the relationship between the wavelength of the Min waves (*WL*) and *d* for the transition to single Min waves in a smaller cell‐size space was investigated. It has been shown that the slower diffusion rate of proteins shortens *WL* in the bulk. Because BSA at concentrations over 50 mg/mL slows down the diffusion rate of proteins in the cytosol of ACs [19], we tested BSA concentrations of 50 mg/mL or more (100, 200, 300, and 350 mg/mL). The experiment showed that the number of Min wave peaks increased with increasing BSA concentration for the same space size. In other words, the slope of the peak numbers per space size, corresponding to 1/*WL*, was larger under slow diffusion conditions (Figure 1B, Figure S2A). Single Min waves were observed in ACs below a certain *d*. Using linear regression of the number of wave peaks against space sizes in the case of ACs with multiple Min waves, we calculated the transition size to single Min waves (*d*_s_). The scatter plot shows that *d*_s_ is proportional to *WL* (Fig. S2B). The relationship between *WL* and π*d*_s_ (corresponding to the circumference of ACs at *d *= *d*_s_) is well explained by the regression of *y* = *x* (*R* ^2^ = 0.81, Figure S2B), indicating that the Min waves become single waves when the circumference of the ACs is smaller than their inherent wavelength.

The results above indicate that some characteristics of single Min waves vary with the space size. To investigate this, we focused on the bandwidth of the wave peaks (*Bw*), which is the macroscopic shape of the Min waves (Figure S2C). The scatter plots of *Bw* against *d* show an obvious difference between single and multiple waves. In the case of multiple Min waves, no significant correlation was observed between *Bw* and *d* (Figure S2C, blue points). In contrast, the *Bw* of the single Min waves showed a strong correlation with *d* (Figure S2C, orange points). Upper limit of the scaling was *d* ~ 25 µm (Figure S2).

Single Min waves showed another remarkable characteristic: the similarity of the wave movement in ACs of various sizes (Figure 1C, Movie S3). The line profiles of sfGFP‐MinD localization on the membrane clearly demonstrated this characteristic. Although the line profiles varied among ACs, line profiles of normalized *d* and fluorescence intensities showed very similar shapes (Figure 1D). Furthermore, kymographs of Min waves in ACs of various sizes suggested that Min wave velocity was proportional to *d* (Figure 1E). The scatter plot of *Bw* and wave velocity against *d* showed a linear relationship with *d* (Figure 1F), indicating size‐dependent scaling of the macroscopic shapes and movement of single Min waves.

The finding of size‐dependent scaling of single Min waves raises the question: What is the factor causing this scaling? We surveyed this factor by changing the parameters involved in scaling single Min waves. It has been shown that reaction and diffusion rates can be modulated by the K^+^ concentration [35], lipid composition [35], and molecular weight of the proteins (Figure S3A). Thus, we first investigated the effects of these parameters on the scaling of the single Min waves.

**FIGURE 2:**
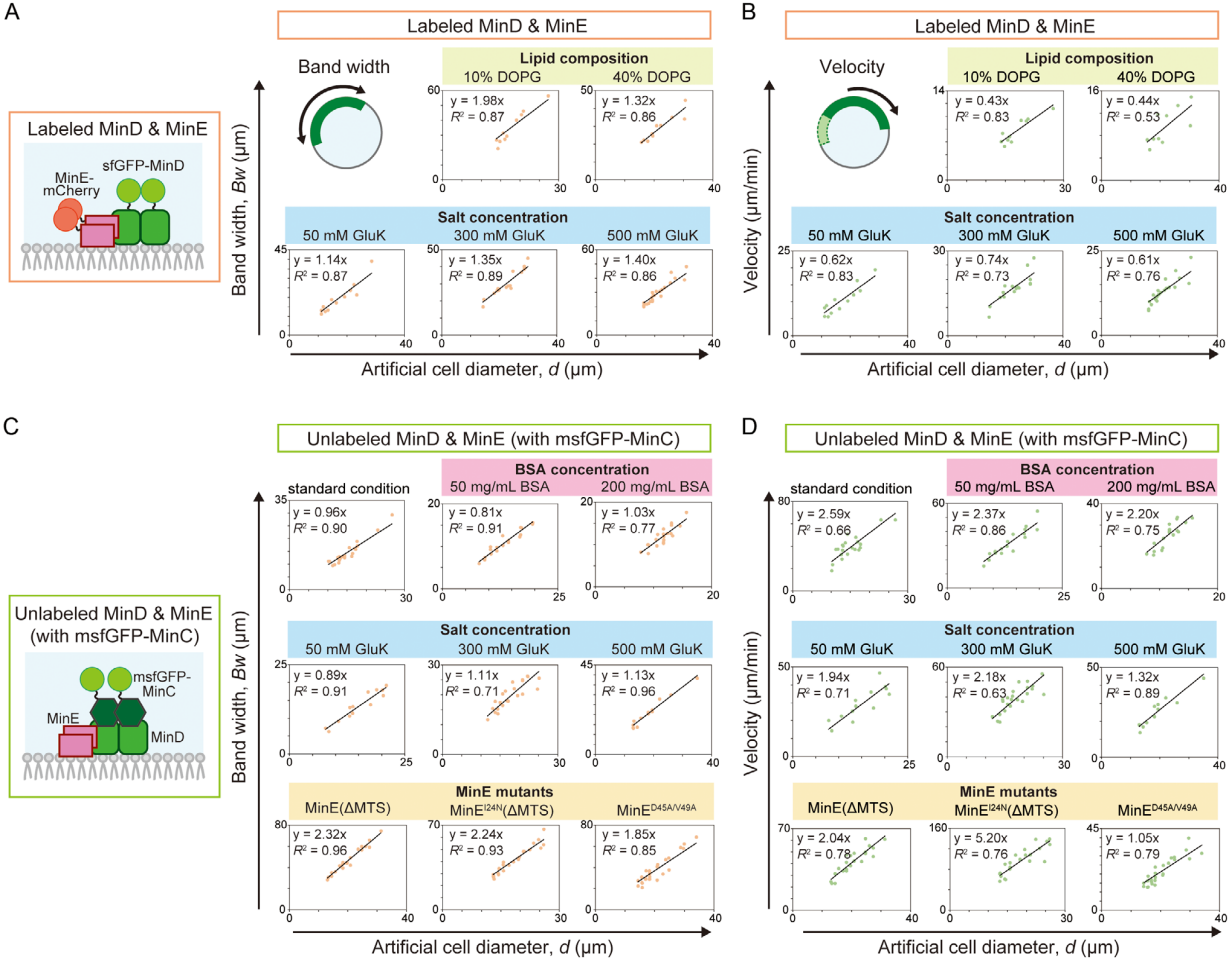
Scaling behaviors of single Min waves under various physicochemical parameters. (A–D) Bandwidth (A, C) and velocity (B, D) of single Min waves in microdroplets under different physicochemical and biochemical conditions are plotted against artificial cell diameter. Linear regressions with zero intercepts are shown. (A, B) Schematic illustration of the experimental system of A and B is shown on the left of A. The standard condition for Min wave generation includes encapsulation of 1 µM sfGFP‐MinD, 1 µM MinE‐mCherry, and 100 mg/mL BSA with 150 mM GluK in microdroplets surrounded by *E. coli* polar lipids. In the top panels of the graphs, *E. coli* polar lipids are replaced with mixtures of DOPE and DOPG at different ratios (10% or 40% DOPG). In the bottom panels, GluK concentrations are varied (50, 300, or 500 mM) (*n* = 10–23 artificial cells). (C, D) Min waves are generated using 1 µM each of unlabeled MinD and MinE, with 0.1 µM msfGFP‐MinC under either 50 or 200 mg/mL BSA (top) and GluK concentrations of 50, 300, or 500 mM (middle). In the bottom panels, Min wave behavior is analyzed using MinE mutants, where 1 μM MinE is replaced by either 0.3 μM MinEΔMTS, 0.3 μM MinE^I24N^ΔMTS, or 1 µM MinE^D45A/V49A^ (bottom) (*n* = 13–26 artificial cells)_._.

K^+^ concentrations varied in the range of 50–300 mM, which is the concentration range in which *Bw* and the velocity of Min waves change sensitively [35]. The lipids for the ACs were changed to mixtures of neutral lipids (DOPE) and anionic lipids (DOPG). The molecular weights of Min proteins (MinD and MinE) were decreased by removing the fluorescent proteins fused with the proteins. In this case, the Min wave was tracked by msfGFP‐MinC, which co‐localizes with ATP‐bound MinD. Under all conditions tested, size‐dependent scaling of single Min waves was also observed (Figure 2, Figure S3B,C).

To further investigate this, we used MinE mutants that affect a broader range of parameters, such as MinE membrane binding, MinDE complex formation, induction of ATPase reactions, and MinE conformational changes [36, 37] (Figure S3A). As a result, the size‐dependent scaling was observed in all MinE mutants we tested (MinEΔMTS, MinE^I24N^ΔMTS, MinE^D45A/V49A^, and MinE^D45A/V49A/I74M^), indicating that its irrelevance to the strength of interaction between lipid membranes and MinE or between MinD and MinE (Figure 2C,D, Figure S3B,C). Moreover, the level of macromolecular crowding caused by BSA (Figure 2C,D) and the lateral diffusion rate of proteins on the membrane (Figures S2,S4) were irrelevant to scaling. Moreover, Min wave generated after artificial cell formation by using ATP regeneration system (Figure S5), indicating the scaling does not depend on initial conditions. These results showed that the size‐dependent scaling of *Bw* and the velocity of the Min wave were independent of the biochemical and physicochemical parameters.

**FIGURE 3:**
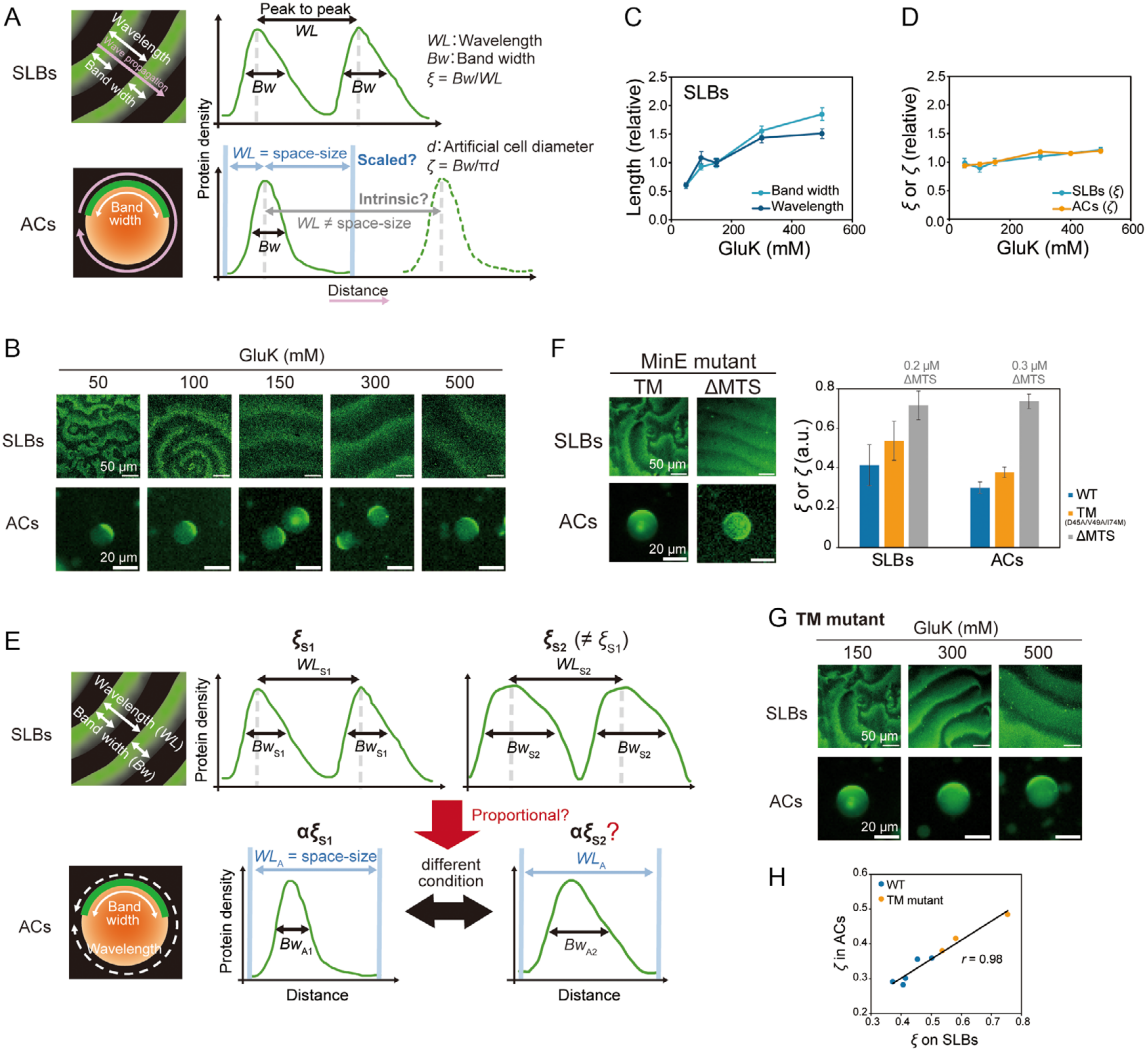
Spatial size of ACs scales the wavelength of single Min waves. (A) Schematic illustration defining the bandwidth (*Bw*) and wavelength (*WL*) of Min waves. A line profile of MinD density along the pink arrow is shown in the right panel. Min waves in artificial cells (ACs) may exhibit either an intrinsic wavelength (gray arrow) or a wavelength that scales with spatial size (light blue arrows). (B–D) Min waves were reconstituted on SLBs and in ACs using 0.1 µM msfGFP‐MinC, 1 µM MinD, and 1 µM MinE under various GluK concentrations. (B) Representative fluorescence images. (C) *Bw* and *WL* of Min waves on SLBs plotted against GluK concentrations (*n* = 10–17). (D) *ξ* = *Bw*/*WL* of Min waves on SLBs and *ζ* = *Bw*/π*d* of Min waves in ACs plotted against GluK concentrations (SLBs; *n* = 10–17, ACs; *n* = 10–23). (E) Schematic illustration of how Min wave shape responds to changes in physicochemical parameters. On SLBs, *Bw*, *WL*, and *ξ* can vary depending on conditions (top). If *ζ* in ACs is functionally equivalent to *ξ* on SLBs, then *ζ* should scale as α*ξ* (bottom). (F) Min waves generated using 0.1 µM msfGFP‐MinC, 1 µM MinD, and 2.5 mM ATP with MinE mutants: 0.7 μM MinE^D45A/V49A/I74M^ (TM mutant) on SLBs and in ACs and MinEΔMTS (0.3 μM for ACs and 0.2 μM for SLBs). Representative fluorescence images (left) and *ξ* on SLBs versus *ζ* in ACs (right) are shown (SLBs; *n* = 16–23, ACs; *n* = 14–22). (G) Fluorescence images of Min waves generated with 0.1 µM msfGFP‐MinC, 1 µM MinD, and 0.7 µM TM mutant of MinE. (H) Correlation between *ξ* on SLBs and *ζ* in ACs. Data from Min waves with wild‐type and TM mutant MinE under various GluK concentrations are shown. (C, D, F, H) For all AC experiments, 100 mg/mL BSA was added. All plots show mean ± standard error.

The fundamental value of spatially periodic patterns generated by RD waves is their wavelength. However, it is not easy to define the wavelength of a single Min wave because it is determined by the length between the wave peaks. The wavelength of a single Min wave can be defined in two ways (Figure 3A). The first is the intrinsic wavelength, which is defined as the distance between multiple waves in bulk. In this case, the wavelength should be constant irrespective of the space size (Figure 3A). The other is the scaled wavelength, which is selected by the space size because single RD waves can be considered multiple waves if periodic boundary conditions are applied (Figure 3A).

To determine which of these two definitions is appropriate, we focused on that K^+^ concentration varies the wavelength of Min waves in bulk. As shown in a previous study [35], in the bulk case, both the wavelength and *Bw* of the Min waves varied with the concentration of K^+^ (Figures 3B, and S6A). Although these are largely varied, we found that their ratio (*ξ* = *Bw*/*WL*) is less affected against K^+^ concentrations (Figure 3B, C,D). By focusing on this property of *ξ*, we evaluated whether the wavelength of a single Min wave is intrinsic or scales with spatial size. If the wavelength were intrinsic, the ratio of *Bw* to π*d* (*ζ* = *Bw*/π*d*) is independent of the *d* of ACs but would vary with K^+^ concentration. In contrast, if the wavelength scaled with *d*, *ζ* would remain robust across different K^+^ concentrations. Our experiments revealed that *ζ* remained nearly constant regardless of K^+^ concentration and closely resembled the profile of *ξ* in bulk (Figure 3B,D), suggesting that spatial size determines the wavelength.

Next, we confirmed the relationships between *ξ* and *ζ* (Figure 3E). In spite of that *ξ* and *ζ* are less affected by space size and K^+^ conditions, *ξ* in bulk is not constant and is varied under biochemical or physicochemical conditions (Figure 3E). In the case of TM mutant (MinE^D45A/V49A/I74M^), although its overall properties are similar to the wild‐type MinE [36, 37], their *ξ* are 1.3 times higher than those of the wild‐type MinE (Figure 3F). Also, ΔMTS mutant, in which membrane targeting sequences of MinE was deleted, *ξ* is two times larger than that of the wild‐type (Figure 3F). These are also the case of *ζ* (Figure 3F). Scatter plotting *ζ* of two MinE mutants at various K^+^ concentrations showed a strong correlation (Figures 3G, H, and S6B), indicating the equivalency between *ζ* and *ξ*. These results support the notion that space size selects the wavelengths of single Min waves.

The size‐dependent scaling of the wavelength indicates why the space sizes scale the Min wave velocity. The periods of the waves were determined by the wavelength/velocity. If both wavelength and velocity were scaled by *d*, their ratio (periods of Min waves) was constant irrespective of *d*. Our experiments showed that the periods of Min waves in ACs showed no correlation with *d* (Figure S6C).

Finally, we analyzed a theoretical model to clarify the origin of size‐dependent scaling of Min waves. We used a model consisting of coupled bulk and surface concentration fields. The model reproduced the experimental wave patterns and wave mode transitions (Methods). The reaction‐diffusion models with the bulk‐surface coupling have been studied mainly in stationary patterns [16, 38]. Recently, the bulk‐surface coupling for the oscillatory waves has been studied to understand the wave generation in the Min system [19, 32, 39]. Although the spatially periodic oscillatory waves, such as traveling and standing waves, can emerge for three or more species [40, 41], their size dependence, particularly under the bulk‐surface coupling, has not yet been elucidated.

**FIGURE 4:**
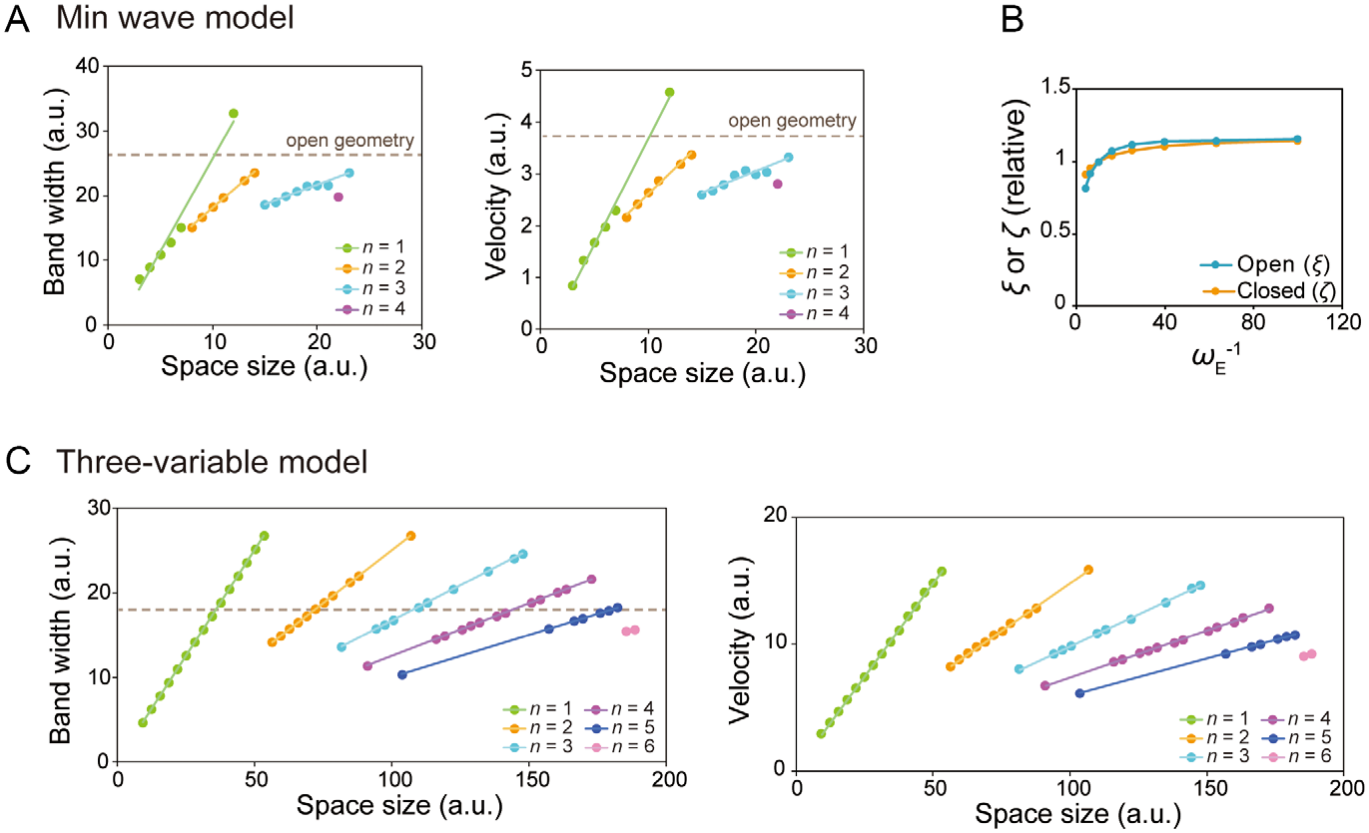
Simulation results for scaling of reaction–diffusion waves in confined spaces. (A) Bandwidth (left) and velocity (right) at each wavenumber (*n*) in the Min wave model plotted against the spatial size of the closed system. Dashed lines represent values from simulations in open systems. (B) *ξ* for the open system and *ζ* for the closed system, obtained from simulations using the Min wave model, plotted against *ω* *_E_* ^−1^. (C) Bandwidth (left) and velocity (right) in the simplified reaction–diffusion model plotted against spatial size (*L*), as in (A). The dashed line indicates the theoretical bandwidth in the limit of infinitely large space.

We numerically solved the reaction‐diffusion equations and analyzed the *WL*, *Bw*, and wave velocity. Consequently, multiple waves appear when *d* exceeds a certain value (Figure 4A) as found in experiments (Figure 1B). Both the *Bw* and wave velocity of the first mode were scaled by size (Figure 4A), recapitulating the experimental results (Figure 1D‐F). We also studied an open system in which we used a square domain with L=256 and H=256. We varied the parameter $\omega_{E}$ (MinE affinity to the lipid membrane) to imitate the K^+^ concentration effect. The result shows that *ξ* is less affected by the change of the parameter (Fig. S6). Similarly, we assessed *ζ* by varying $\omega_{E}$, and confirmed that both *ζ* and *ξ* are less affected by system sizes (Figure 4B), corresponding to the experimental results (Figure 3 C,D). These results clearly show that the wavelength selection of Min waves by space size can be explained by the reaction‐diffusion model of Min waves.

To see the generality of size‐dependent scaling, we demonstrated numerical simulations of a simple model consisting of three variables U(x,t),V(x,t),W(x.t), defined on a 1D periodic space of size L (see Theoretical model: Three‐variable model in Methods). This three‐variable model was built to express dynamic waves (traveling or standing) emerge when uniform oscillatory instability (Hopf bifurcation) is suppressed by confinement effects [19]. Because Min wave model used above is complex due to six variables with bulk and surface coupling, it does not bring clear reason why wavelength selection of Min waves by space sizes. Therefore, we examined whether the size‐dependent scaling recapitulated by this simplified three‐variable model. To degenerate the Min wave model, we considered a two‐variable activator‐inhibitor‐type reaction‐diffusion equation.

Here, *U* and *V* correspond to oscillatory membrane dynamics, whereas *W* indicates bulk dynamics. Even in this simple model, *Bw* and wave velocity were scaled by system size (Figure 4C), indicating that size‐dependent scaling is not specific to Min waves but is general among iRD waves.

Although iRD waves are a fundamental mechanism for determining positional information in living cells, the reason why they are generally found in cell size confinement remains elusive. In this study, by using the reconstitution system of Min waves in cell‐size confinement, we found that the wavelength of single iRD waves was selected by space size, and the wavelength selection confers responsibility to iRD waves among various cell sizes. Our experimental and theoretical analyses showed that wavelength selection by confinement was observed irrespective of the parameters for reactions and diffusion and was a general characteristic among iRD waves (Figure 4).

A plausible explanation for the size‐dependent scaling of the iRD waves is the selection of a periodic solution of the RD equations. In a 1D closed space of length *L*, the concentration *c*(*x*) of the molecules at position *x* is *c*(*x+L*) = *c*(*x*) because of the periodic boundary conditions of the closed space. Simultaneously, a periodic solution of RD equations with wavelength *λ* is denoted as *c*(*x+λ*) = *c*(*x*). In the case of a single wave, *L* = *λ* is necessary for satisfying these two conditions. However, because *L* should be constant, only *λ* can be varied. Consequently, *λ* should be scaled by the space size, *L*. This explanation implies that the wavelength selection of RD waves by space size is a universal principle for general RD systems with periodic patterns.

Previous theoretical studies of intracellular reaction‐diffusion systems have focused on macroscopically stationary patterns (Turing pattern). By incorporating the effects of confined geometry and/or bulk surface coupling, these studies have clarified various characteristics of Turing patterns, including the conditions for pattern formation, the sensitivity of wave numbers and peak positions to initial and boundary conditions [16, 42, 43]. In this study, we investigated the characteristics of an iRD wave (Min wave) in the cell‐scale space. In the case of iRD waves, bulk‐surface coupling effectively changes reaction parameters through changes in steady‐state concentration derived from spatial sizes. Therefore, the impact of variations in spatial size and physicochemical parameters on the shape and speed of iRD waves were largely elusive, both theoretically and experimentally. By combining a controlled, quantitative experimental system and theoretical analyses, we have derived how iRD wave responds to spatial and physicochemical fluctuations. Our analysis reveals that even under confinement and bulk‐surface coupling, a single wave much smaller than the wave width for the bulk systems maintains its wave shape (e.g., bandwidth) remarkably robustly by scaling their wavelength with spatial size. Moreover, we confirmed that, in response to fluctuations in reaction parameters such as salt concentration, while the wave speed changes, the wave shape of single waves is maintained. This combination of shape robustness and velocity plasticity is a novel property of iRD waves that have not been discussed within existing theoretical frameworks.

The mechanism by which Min waves with wavelengths larger than the size of a cell are entrapped within confined spaces has been discussed (wavelength problem) [13, 44]. One explanation is that the wavelength of Min waves in living cells is shortened by biochemical factors that are missing owing to the reconstituted system or physicochemical factors [35, 45], such as macromolecular crowding in cells. Genetic interactions suggest that some biochemical factors are missing [46]; however, these factors have not yet been identified. Another explanation is that the nontrivial property of Min waves results from the solution of a nonlinear equation far from equilibrium conditions. Although this explanation suggests the importance of small, confined spaces for iRD waves [44, 47, 48], it does not provide a mechanism for how the bulk surface ratio regulates the wavelength. Wavelength scaling demonstrated in this study has been less appreciated but can serve as a third perspective for solving wavelength problems.

The size‐dependent scaling of iRD waves found in this study provides biological insights into why cells use them, despite their complexity, for spatiotemporal determination systems. First, scaling is effective in maintaining the relative position information within cells, irrespective of cell size changes during development and proliferation. This is an unexplored benefit of iRD waves for intracellular molecular placement, following the previous finding that a non‐scaling reaction‐diffusion mechanism sets the threshold of the space size to form cell polarity [49], although it belongs to Turing pattern rather than iRD waves. Studies on actin waves in amoebae and Cdc42 localization in budding yeast also showed scaling properties based on the space size of living cells, although the mechanism have been still elusive [50, 51]. The universal characteristics of the iRD waves observed in this study provide theoretical and experimental support for this hypothesis. Second, the scaling of iRD waves is beneficial for robustness to environmental shifts. With environmental shifts, K^+^, which is the most abundant cation in living cells, changes easily [52]. As shown here, wavelength selection suppressed this change, allowing the patterns to be maintained in the cell‐size space regardless of the K^+^ concentration. In general, wavelength scaling shows that iRD waves are responsive for determining positional information within cells, regardless of changes in cell size or environmental shifts.

## 3 Conclusion

This study demonstrates that spatial confinement is a critical physical parameter that drives the size‐dependent scaling of iRD waves with wave selection, and this scaling ensures robust spatiotemporal patterning within cell‐sized compartments. The elucidation of this coupling between space size and RD wave dynamics in small space indicates a fundamental mechanism for how living cells achieve reliable spatiotemporal regulation against variations in spatial and physicochemical fluctuations. Although this study uses a reconstitution system of an iRD wave for simplification, actual living cells are complex, including their elements and shapes. In this regard, behavior similar to the Min wave scaling has also been reported in experiments using a mold‐shaped like *E. coli* [53, 47]. It should be explored how more complex and biologically relevant shapes of spaces influence the scaling and stability of iRD waves in the future, which will be essential for a comprehensive understanding of self‐organization across the diverse morphologies found in living systems.

## 4 Experimental Section

### 4.1 Expression and Purification of Min Proteins

His‐sfGFP‐MinD, His‐msfGFP‐MinD, MinE‐mCherry‐His, His‐msfGFP‐MinC, His‐MinD, and MinE‐His were overexpressed in *Escherichia coli* cells and purified as described in our previous studies [19, 26]. Briefly, *Escherichia coli* BL21‐CodonPlus(DE3)‐RIPL cells (Agilent Technologies, Santa Clara, CA, USA) were transformed with either pET15‐sfGFP‐MinD, pET15‐msfGFP‐MinD, pET29‐MinE‐mCherry‐His, pET15‐msfGFP‐MinC, pET15‐MinD, or pET29‐MinE‐His. Cells were cultivated in LB medium at 37°C with 100 μg/mL ampicillin (His‐sfGFP‐MinD, His‐msfGFP‐MinD, His‐msfGFP‐MinC, and His‐MinD) or 50 μg/mL kanamycin (MinE‐mCherry‐His and MinE‐His). Proteins were overexpressed by the addition of 1 mM IPTG at OD_600_ = 0.1–0.2 (His‐sfGFP‐MinD, MinE‐mCherry‐His, His‐msfGFP‐MinC, and MinE‐His) or 0.8 (His‐msfGFP‐MinD and His‐MinD). Cells were further cultivated at 37°C (all except MinE‐mCherry‐His) or 16°C (MinE‐mCherry‐His) for 1.5 h (His‐msfGFP‐MinD and His‐MinD), 3 h (His‐sfGFP‐MinD, His‐msfGFP‐MinC, and MinE‐His), or 12 h (MinE‐mCherry‐His). After the cultivation, cells were harvested by centrifugation at 8000 × g at 4°C for 2 min. In the case of His‐sfGFP‐MinD, His‐msfGFP‐MinC, MinE‐His, and MinE‐mCherry‐His, cells were resuspended in the lysis buffer [50 mM tris‐HCl (pH 7.6), 300 mM NaCl, 1 mM phenylmethylsulfonyl fluoride (PMSF), 10 mM imidazole, 1 mM DTT, and 0.1 mM ADP for MinD] and sonicated by using Sonifier250 (Branson, Danbury, CT, USA). The crude extracts were centrifugated at 20 000 × g at 4°C for 30 min. The supernatants were filtered by HPF Millex HV (Merck Millipore, Billerica, MA, USA) and mixed with cOmplete His‐Tag Purification Resin (Roche, Basel, Switzerland). The mixture gently shaken for 30 min at 4°C were loaded into Poly‐Prep Chromatography Column (Bio‐Rad, Hercules, CA, USA). The resin was washed by 25 mL of the wash buffer [50 mM tris‐HCl (pH 7.6), 300 mM NaCl, 1 mM PMSF, 0.1 mM EDTA, 20 mM imidazole, and 10% glycerol], and His‐tagged proteins were eluted by adding 2 mL of elution buffer [50 mM tris‐HCl (pH 7.6), 300 mM NaCl, 1 mM PMSF, 0.1 mM EDTA, 250 mM imidazole, and 10% glycerol]. Elution buffer was exchanged with the storage buffer [50 mM Hepes‐KOH (pH 7.6), 150 mM GluK, 0.1 mM EDTA, 10% glycerol, and 0.1 mM ADP for MinD] by using AmiconUltra‐0.5 3K (MinE‐His) or 10K (His‐msfGFP‐MinD and His‐msfGFP‐MinC) (Merk Millipore). His‐msfGFP‐MinD and His‐MinD were purified by similar procedures by using the lysis buffer containing 20 mM imidazole and 0.1 mM ADP, Ni Sepharose 6 Fast Flow (Cytiva, Tokyo, Japan), Wash buffer containing 25 mM imidazole, and the elution buffer containing 500 mM imidazole. For MinE‐mCherry‐His, the eluted fraction was diluted 5‐ to 10‐fold with HG buffer [50 mM Hepes‐KOH (pH 7.6), 10% glycerol, and 0.1 mM EDTA] and further purified by using HiTrap Q HP column (GE Healthcare, Chicago, IL, USA) and AKTA start (GE Healthcare). The diluted solution was loaded into the column equilibrated with A buffer [50 mM HEPES‐KOH (pH 7.6), 50 mM NaCl, 10% glycerol, and 0.1 mM EDTA], and the column was washed with the same buffer. The proteins were fractionated by using the IEX protocol of AKTA start using A buffer and B buffer [50 mM HEPES‐KOH (pH 7.6), 1 M NaCl, 10% glycerol, and 0.1 mM EDTA]. Peak fractions monitored by SDS‐PAGE were collected and exchanged to the storage buffer using AmiconUltra‐15 10 k (Merck Millipore) and AmiconUltra‐0.5 10 k filters (Merck Millipore).

To construct pET29‐MinEΔMTS‐His, pET29‐MinE^I24N^ΔMTS‐His, pET29‐MinE^D45A/V49A^‐His, and pET29‐MinE^D45A/V49A/I74M^‐His, the DNA fragments were amplified from pET29 plasmids containing each MinE mutant and mCherry‐His constructed in our previous study [54] by PCR using KOD One DNA polymerase (TOYOBO) and oligo primers. DNA fragments amplified by PCR treated with DpnI were ligated by the iVEC3 method [55]. Each MinE mutant was expressed and purified by the similar procedures to His‐MinD using kanamycin instead of ampicillin for cultivation and ADP was omitted from both Lysis buffer and Storage buffer. Concentrations of all purified proteins were estimated by BCA assay or the band intensities after CBB staining of SDS‐PAGE gels using Fiji software (National Institutes of Health, Bethesda, MD, USA).

### 4.2 Self‐Organization Assay Inside Microdroplets Covered With Lipids

Microdroplets covered with *E. coli* polar lipids [54] were used as the standard experimental condition. *E. coli* polar lipids in chloroform (25 mg/mL) (Avanti, Alabaster, AL, USA) was dried by gentle flow of argon gas in glass tubes, and mineral oil (Nacalai Tesque, Kyoto, Japan) was added to a lipid concentration of 1 mg/mL. To dissolve the lipid in oil, the mixture was sonicated for 90 min at 60°C using Bransonic (Branson) and mixed by vortexing for 1 min.

The mixture, 1 μM His‐sfGFP‐MinD, 1 μM MinE‐mCherry‐His, 2.5 mM ATP, and 100 mg/mL BSA in the reaction buffer [25 mM Tris‐HCl (pH 7.6), 150 mM GluK, and 5 mM GluMg] were prepared as an inner solution. BSA solution was prepared by dissolving BSA (A6003, Sigma–Aldrich, St. Louis, MO, USA) in water and washing with the reaction buffer by using AmiconUltra‐0.5 50K (Merck Millipore). BSA concentrations were estimated by Pierce BCA Protein Assay kit (Thermo Fisher Scientific, Waltham, MA, USA). Microdroplets were obtained by adding 2 μL of the inner solution to 100 μL of the lipid–oil mixture and by tapping. The microdroplet solution was transferred into the slit between two cover glasses. The behavior of Min proteins within the microdroplets was observed by observed using a fluorescence microscope (AxioObserver Z1; Carl Zeiss, Jena, Germany) with 10 or 20 s intervals. All time‐lapse images obtained were processed and analyzed by using Fiji software.

The compositions of lipids and inner solution were varied if indicated. DOPG was mixed with DOPE or DOPC in chloroform (Avanti) at 10–40 mol%, and chloroform was dried by gentle argon gas flow. For the mixture of DOPC and DOPG, the lipid films were further dried in a vacuum desiccator for 1.5 h. Then, lipid‐oil mixtures were similarly prepared as *E. coli* polar lipids. Concentrations of GluK as salt and BSA in the inner solution was also varied if indicated. In the case of unlabeled His‐MinD and MinE‐His, labeled MinD and MinE were replaced with 1 μM His‐MinD, 1 μM MinE‐His. In this case, 0.1 μM His‐msfGFP‐MinC was added to the mixture for tracking Min waves. In the case of the investigation of MinE mutants to the scaling of Min wave, either 0.3 μM MinEΔMTS‐His, 0.3 μM MinE^I24N^ΔMTS‐His, 1 μM MinE^D45A/V49A^‐His, or 0.7 μM MinE^D45A/V49A/I74M^‐His was mixed with 0.1 μM His‐msfGFP‐MinC, 1 μM His‐MinD, 2.5 mM ATP, and 100 mg/mL BSA in the reaction buffer.

### 4.3 Self‐Organization Assay of Min Proteins on SLBs

SLBs were prepared by the following protocols modified based on a previous study [56]. The reaction chamber was obtained by attaching the upper rim of a 0.2 mL tube cut off its lid and the bottom to the cover glass by using UV‐glue (NOA63, Norland Products, Cranbury, NJ) and exposing to 360 nm light for 1 min. The chamber was placed in ion bombarder PIB‐10 (Vacuum Device, Ibaraki, Japan) and hydrophilized by HARD mode for 3 min.

To obtain SUVs for the supported lipid bilayer formation, 100 μL of *E. coli* polar lipids in chloroform (25 mg/mL) were poured into a glass tube and were dried by argon gas flow and incubation in a vacuum desiccator for 3 h. To prepare multilamellar vesicles (MLVs), TK150 buffer [25 mM Tris‐HCl (pH 7.6) and 150 mM KCl] was added to the final lipid concentration of 4 mg/mL. After overnight incubation at room temperature, MLVs were formed by vortex for 1 min. MLVs were converted to small unilamellar vesicles (SUVs) by extrusion using Avanti Mini Extruder (Avanti) and the PC membranes with pores of 1.0, 0.4, and 0.05 μm (Avanti) in this order.

SUVs solutions were diluted by TK150 buffer to 2 mg/mL of lipids, and then, 1 mM CaCl_2_ at final was added to the solution. The mixture was transferred to the reaction chamber prepared above and incubated for 5 min at room temperature. Then, the solution was removed and washed by TK150 buffer for four times to remove excess SUVs and CaCl_2_. Then, TK150 buffer was replaced with the reaction buffer by the cycle of the addition and removal of the reaction buffer for five times.

For self‐organization assay of Min waves, 0.1 μM His‐msfGFP‐MinC, 1 μM His‐MinD, 1 μM MinE‐His, and 2.5 mM ATP was added into the chamber to the indicated final concentration. After the incubation for 2 h at room temperature, Min waves were observed by using fluorescence microscope (AxioObserver Z1) or confocal laser scanning microscope FV1000 (Olympus, Tokyo, Japan).

### 4.4 Diffusion Analysis

The lipid–oil mixture containing *E. coli* polar lipids, DOPE and DOPG, or DOPC and DOPG was prepared as described above. Microdroplets encapsulating 1 μM His‐msfGFP‐MinD, 2.5 mM ATP, and 100 mg/mL BSA in reaction buffer were prepared by tapping of the mixture added to the lipid solution. Diffusion coefficient of His‐msfGFP‐MinD bound on the membrane was analyzed by fluorescence recovery after photo‐bleaching (FRAP) using tornado bleaching of FV1200 (Olympus). To obtain diffusion coefficients, the FRAP protocol of FV1200 was used.

### 4.5 Analysis of Min Waves

To measure the bandwidth of the Min wave, the diameter of the ACs and the angle formed by both ends of the Min wave were generated on the cross‐section of the artificial cell and the center of the artificial cell using Fiji software. The band width was calculated as (artificial cell diameter) × *π* × (central angle of the Min wave band)/360°. To obtain the velocity of the Min wave, the angle and time at which the Min wave propagated more than once in the artificial cell were measured. Before calculating the velocity, the period of the Min wave was obtained as (time) × 360°/ (propagating angle). Then, the velocity was calculated as (artificial cell diameter)×*π* / (period).

### 4.6 Theoretical Model: Min Model

Numerical simulations of the model were performed based on previous studies [19, 26, 32]. Among various models that represent Min waves [32, 33, 57], we chose the model we used in our previous paper [26] because it closely matches the dynamics of Min waves in artificial cells and incorporates both the effect of ATP hydrolysis of MinD [33, 57] and persistent MinE binding [32], which were separately investigated. Because we were interested in the generic properties of size scaling, we focus on 2D closed and open systems. The closed system has a disk‐shaped bulk domain with radius R. The circular boundary of the disk was treated as a membrane. The open system has a rectangular domain with height H, and a flat membrane of size L. In both systems, the ATP‐MinD, ADP‐MinD, and MinE concentrations on the membrane are denoted as $c_{DT}$, $c_{DD}$, and $c_{E}$, respectively. The concentrations of MinD, MinE, and their complex (MinDE) bound to the membrane were denoted as $c_{d}$, $c_{de}$, and $c_{e}$, respectively. We considered reaction‐diffusion equations with the reaction rate ω. Different types of rates are specified by the subscript of ω. Recruitment of MinD onto the membrane from the cytosol is given by $\omega_{D}$ and $\omega_{dD}$, where in the latter reaction, membrane‐bound MinD recruits MinD in the cytosol. Recruitment of MinE in the cytosol by membrane‐bound MinD is described by $\omega_{E}$. The binding rates $\omega_{ed}$ of MinD and MinE to the membrane were assumed to be high. Attachment and detachment of MinE from/to the cytosol are described by $\omega_{eE}$ and $\omega_{e}$, respectively. The rate of MinD dissociation from the membrane is expressed by $\omega_{de,m}$. We assumed the same diffusion constants $D_{m}$ for proteins bound to the membrane, and set them to unity without loss of generality. We also assumed that the bulk diffusion constants of the unbound proteins were the same and denoted them by D. The time scale was normalized using the $1/\omega_{e}$. This model is expressed by the following equation:

(1) $$\partial_{t}\left(c_{DT}+c_{DD}\right)=D\Delta \left(c_{DT}+c_{DD}\right)$$

(2) $$\partial_{t}c_{DD}=D\left(\Delta -\frac{1}{\eta^{2}}\right)c_{DD}$$

(3) $$\partial_{t}c_{E}=D\Delta c_{E}$$

(4) $$\partial_{t}c_{d}=\Delta_{s}c_{d}+c_{DT}\left(\omega_{D}+\omega_{dD}c_{d}\right)-\omega_{E}c_{E}c_{d}-\omega_{ed}c_{e}c_{d}$$

(5) $$\partial_{t}c_{de}=\Delta_{s}c_{de}+\omega_{E}c_{E}c_{d}+\omega_{ed}c_{e}c_{d}-\omega_{de,m}c_{de}$$

(6) $$\partial_{t}c_{e}=\Delta_{s}c_{e}+\omega_{eE}c_{E}+\omega_{de,m}c_{de}-\omega_{ed}c_{e}c_{d}-c_{e}$$

where Δ and $\Delta_{s}$ denote the Laplacian operator in 2D bulk space and the Laplace–Bertrami operator on the 1D membrane, respectively. The boundary conditions in Equation (1) (2013); (3) are as follows:

(7) $$-D\nabla \left(c_{DT}+c_{DD}\right)=c_{DT}\left(\omega_{D}+\omega_{dD}c_{d}\right)-c_{de}$$

(8) $$-D\nabla c_{DD}=-c_{de}$$

(9) $$-D\nabla c_{E}=\omega_{E}c_{E}c_{d}-c_{e}+\omega_{eE}c_{E}$$

The boundary conditions were chosen such that the total amounts of MinD and MinE are conserved. The length scale associated with ATP hydrolyzation is denoted by $\eta =\sqrt{D/\lambda }$ where λ is the ADP/ATP exchange rate. We set λ=1. Equations (1) – (6) were solved using the COMSOL software. We mainly chose the parameters as, *H* = 256, D=100, $\omega_{D}=0.1$, $\omega_{dD}=5.0$, $\omega_{E}=0.1$, $\omega_{ed}=100$, $\omega_{de,m}=1.0$, and $\omega_{eE}=0.04$, and varied $\omega_{E}$ when the dependence on the parameter was discussed in the text. For each parameter set, we performed simulations for time T=2000. The initial condition was chosen as a small gradient in one direction on the membrane with a Gaussian random distribution. After the relaxation time t≥1000, the properties of the wave, such as the wavelength, bandwidth, and frequency, were computed.

Inhomogeneity of the concentration field was expressed by the amplitude of each mode in the expansion of the concentration $c_{d}$ by the Fourier expansion in the polar coordinates (r,θ) as

(10) $$c_{d}(\theta ,t)=\sum_{l=-\infty }^{\infty }c_{d,l}(t)e^{il\theta }$$

For example, the uniform distribution of MinD on a membrane is expressed by the *l* = 0 mode and its norm $\left|c_{d,0}\right|$, whereas the first mode (*l* = 1) corresponds to the inhomogeneous concentration field of a single wave, characterized by the norm $|c_{d,1}|=\sqrt{c_{d,+1}^{2}+c_{d,-1}^{2}}$. We analyzed the waves using the MinD concentration, including both MinD and MinDE, that is, ${\tilde{c}}_{d}=c_{d}+c_{de}$. We evaluated the wavenumber $l^{*}$ by maximum amplitude of the modes $l^{*}=\underset{l\neq 0}{\text{argmax}}|{\tilde{c}}_{d,l}|$. Bandwidth (*Bw*) was evaluated as the total length of the membrane at which the concentration of MinD was greater than half of its maximum concentration on the membrane. The total length was divided by the wavenumber to obtain *Bw*. The frequency of the wave was computed by the time derivative of the phase $\phi (t)=\tan^{-1}{\tilde{c}}_{d,-1}/{\tilde{c}}_{d,1}$ of MinD on the membrane.

### 4.7 Theoretical Model: Three‐Variable Model

We performed numerical simulations of a model consisting of three variables, U(x,t),V(x,t),W(x.t), defined in a 1D periodic space of size L. The model reads

(11) $$\partial_{t}U=\lambda U-\omega V-s_{1}W-c_{3}U^{3}+D_{U}\Delta U$$

(12) $$\partial_{t}V=\lambda V+\omega U-c_{3}V^{3}+D_{V}\Delta U$$

(13) $$\partial_{t}W=\tau^{-1}\left(U-W\right)+D_{W}\Delta W$$

Here, *D*_*U*_, *D*_*V*_, and *D*_*W*_ indicate diffusion coefficients of *U*, *V*, and *W*, respectively. *λ* and *ω* indicate reaction constants. *τ* is a time scale of the third variable, *W*, compared with other two variables, *U* and *V.* We chose the parameters as $\lambda =1,\omega =1,s_{1}=2.0,c_{3}=1.0,\tau =0.01,D_{U}=D_{V}=1.0,D_{W}=1000/\tau$. Setting smaller *τ* indicates that bulk diffusion is fast to keep nearly at steady state like Min waves. Therefore, when $D_{W}\gg 1,\tau \ll 1$, the dynamics of the third variable, W, is fast and is slaved by other two variables. It diffuses faster and exhibits global inhibition owing to $-s_{1}W$ in U equation. The model was solved using custom code written in MATLAB. The wavelength, *Bw*, and frequency were analyzed in the same manner as for the Min model.

## Supporting Information

Additional supporting information can be found online in the Supporting Information section.

## Funding

This work was funded by the Japan Society for the Promotion of Science (JP18H04565, JP20H01875, JP20H047175, JP22H04851, JP22H05432), the Keio University (Ishii‐Ishibashi Fund (The Keio University Grant for Early Career Researchers), the Japan Science and Technology Agency (JPMJFR213Y, JPMJFR2140, JPMJFR2250).

## Conflicts of Interest

The authors declare no conflicts of interest.

## Supporting information

Supplementary Material

## Data Availability

The data, code, and other materials in this study are available from the corresponding author upon reasonable request.
