# Supplementary material for: Cell‐Size Confinement Drives Size‐Dependent Scaling of Intracellular Reaction‐Diffusion Waves for Robust Patterning
Source: Small Sci. 2026 Apr 23;6(4):e202500643. doi: 10.1002/smsc.202500643 (PMC13116265; doi:10.1002/smsc.202500643)
Supplement: Supplementary file 1 — Supplementary Material [file SMSC-6-e202500643-s001.pdf]

Supporting Information

Cell-Size Confinement Drives Size-Dependent Scaling of Intracellular Reaction-Diffusion Waves for Robust Patterning

Sakura Takada †, Shunshi Kohyama †, Miho Yanagisawa, Nobuhide Doi, Natsuhiko Yoshinaga *, Kei Fujiwara *

† These authors contributed equally

* Corresponding authors

Contents:

Supplementary Figure S1. Proportion of Min wave modes and cell size distribution of the Min wave reconstituted in artificial cells (ACs).

Supplementary Figure S2. Effects of BSA Concentration and space sizes on Min waves.

Supplementary Figure S3. Modulation of reaction and diffusion parameters by changing experimental conditions.

Supplementary Figure S4. Diffusion coefficients of msfGFP-MinD on the membrane of ACs.

Supplementary Figure S5. Scaling of band width and velocity of Min waves in artificial cells under the homogenous initial condition.

Supplementary Figure S6. Effects of K^+ concentration and space sizes on Min waves.

Supplementary Figure S7. Bandwidth of Min waves simulated by theoretical model varying ω_E.

Supplementary Movie S1. Multiple Min waves in artificial cells.

Supplementary Movie S2. 4D images of single Min waves in artificial cells.

Supplementary Movie S3. Scaling of Min waves in different sizes of artificial cells.

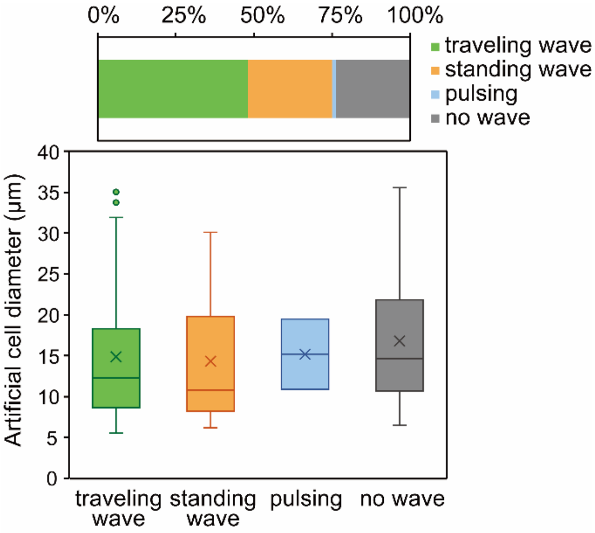

Fig. S1. Proportion of Min wave modes and cell size distribution of Min waves reconstituted in artificial cells (ACs). Bar graphs (top) show the frequency of each Min wave pattern generated by encapsulating 1 μM sfGFP-MinD, 1 μM MinE-mCherry, 2.5 mM ATP, and 100 mg/mL BSA in ACs with $d > 5$ μm (n = 148). Box plots (bottom) display the distribution of AC sizes corresponding to each pattern.

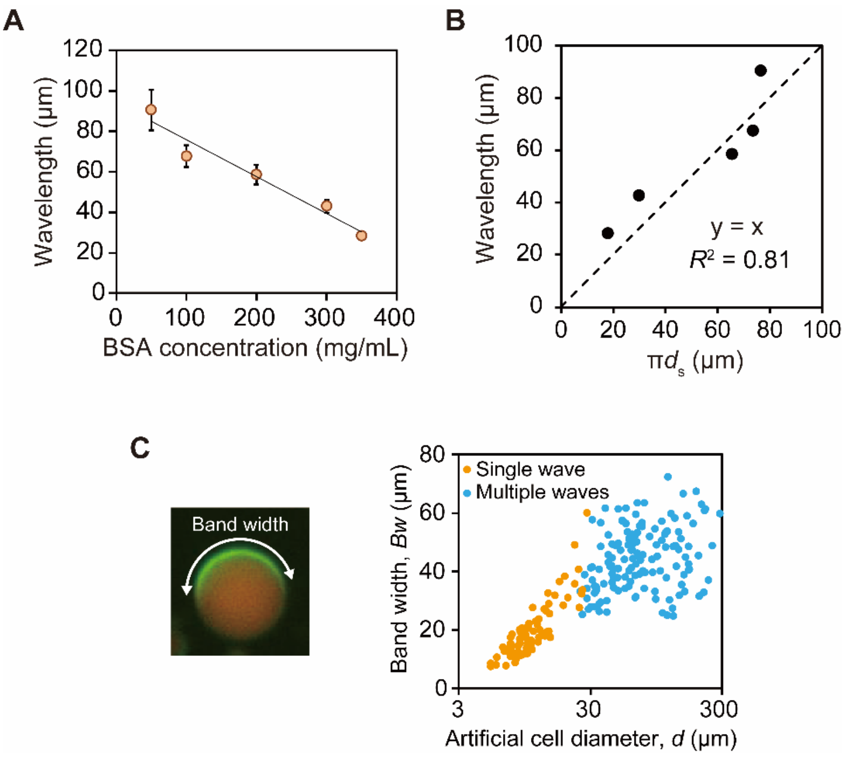

Fig. S2. Effects of BSA concentration and spatial size on Min waves. (A) Relationship between BSA concentration and the wavelength of Min waves in artificial cells. Min waves were generated in artificial cells encapsulating 1 µM sfGFP-MinD, 1 µM MinE-mCherry, 2.5 mM ATP, and varying concentrations of BSA from 50 to 350 mg/mL. (B) Relationship between the transition size of artificial cells to a single wave and the wavelength of Min waves. Wavelengths of Min waves generated under different BSA concentrations are plotted against the circumference of the largest artificial cell exhibiting a single wave. (C) Relationship between spatial size and the bandwidth of Min waves in artificial cells. A white arrow indicates the region corresponding to the bandwidth of a Min wave (left). Bandwidths of single (n = 80) and multiple (n = 142) Min waves are plotted against artificial cell diameter (right).

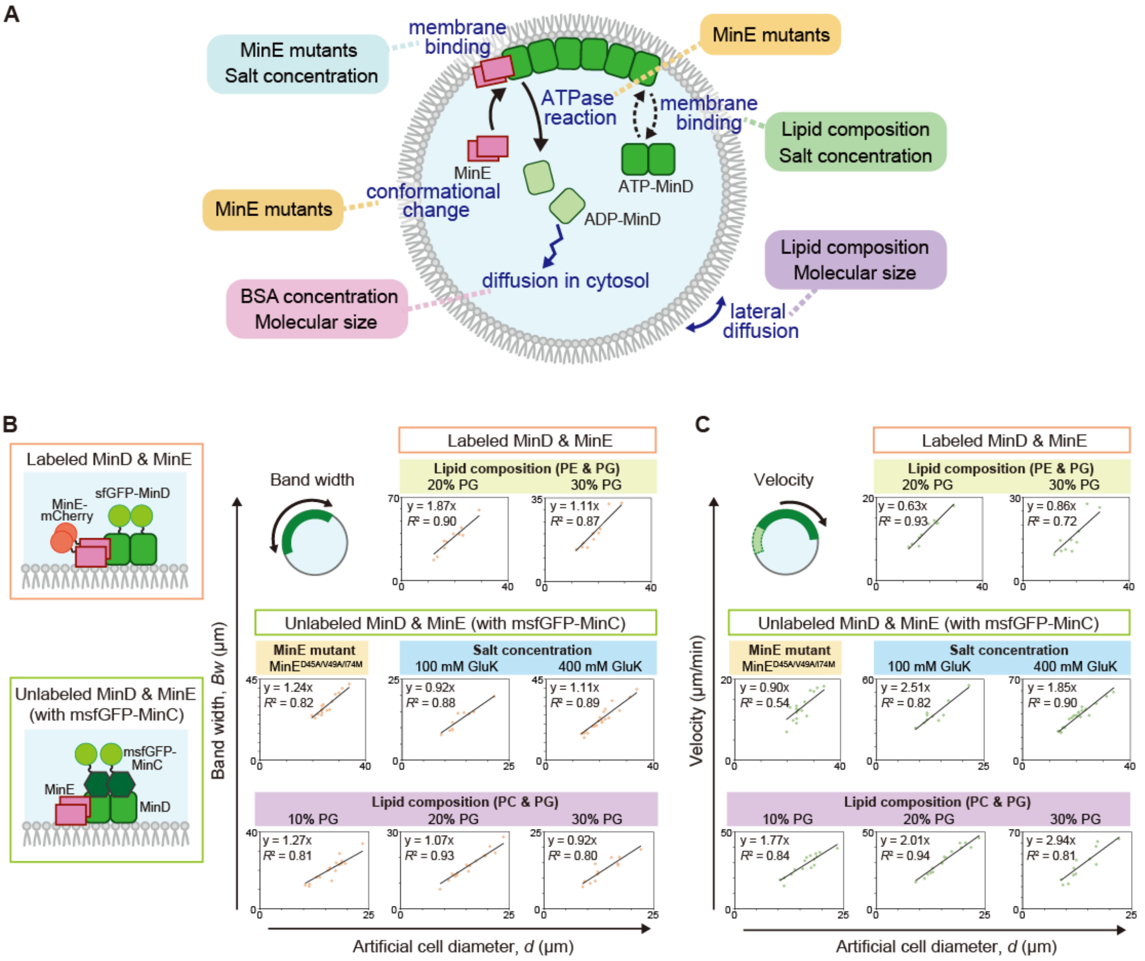

Fig. S3. Modulation of reaction and diffusion parameters by changing experimental conditions. (A) Schematic illustration of the reaction and diffusion parameters involved in Min wave generation, along with the experimental conditions that can modulate these parameters. (B) Scaling of the bandwidth of single Min waves in artificial cells under various conditions. Schematic illustration of the experimental system of B and C is shown in the left. Bandwidths of single Min waves are plotted against artificial cell diameters. The standard condition includes 1 µM sfGFP-MinD, 1 µM MinE-mCherry, 2.5 mM ATP, 150 mM GluK, and 100 mg/mL BSA in droplets covered with *E. coli* polar lipids (top). In the middle and bottom panels, MinDE is replaced with 0.1 µM msfGFP-MinC, 1 µM MinD, and 1 µM MinE. Lipid composition, MinE mutants, and salt concentrations are varied as indicated in the figure labels (n = 10–20 artificial cells). (C) Scaling of the velocity of single Min waves in artificial cells under the same conditions as in panel B. Velocity is plotted against artificial cell diameter (n = 10–20 artificial cells). (B, C) Linear regression lines with zero intercepts are shown.

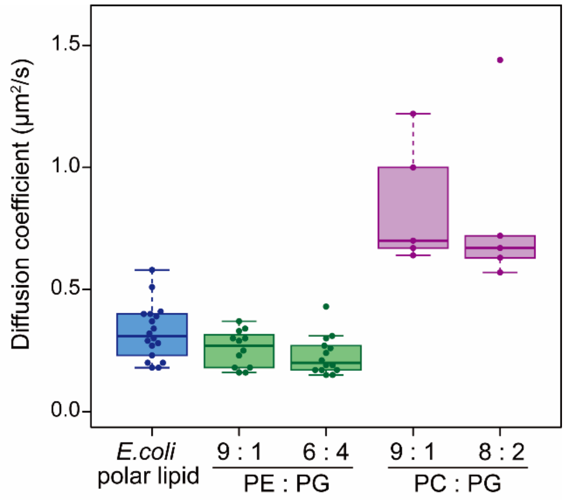

Fig. S4. Diffusion coefficients of msfGFP-MinD on the membrane of ACs. Diffusion coefficients of membrane-bound msfGFP-MinD were measured by FRAP. ACs were prepared by encapsulating 1 μM msfGFP-MinD, 2.5 mM ATP, and 100 mg/mL BSA in droplets covered with either *E. coli* polar lipids, a mixture of DOPE and DOPG, or a mixture of DOPC and DOPG (n = 5–18).

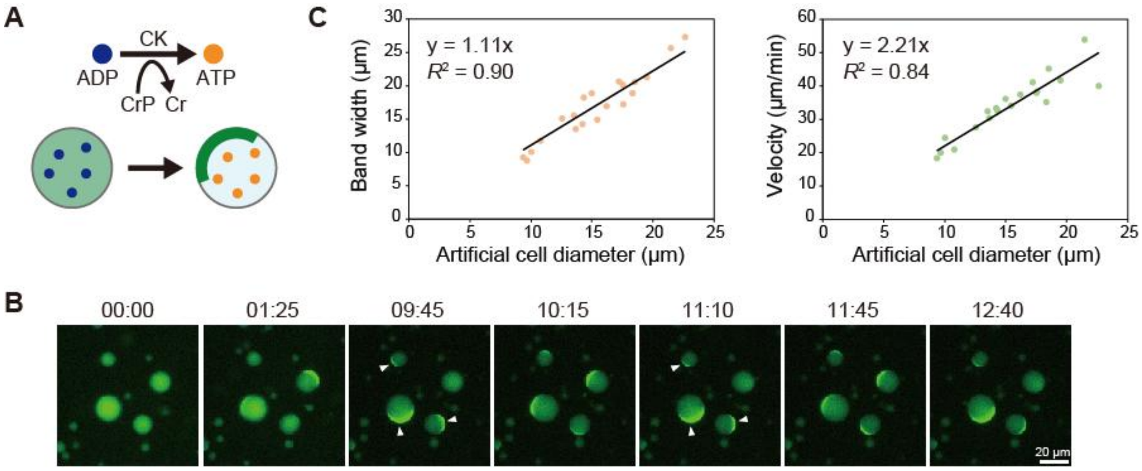

Fig. S5. Scaling of band width and velocity of Min waves in artificial cells under the homogenous initial condition. (A) Schematic illustration of the experimental setup. In addition to Min proteins (1 µM msfGFP-MinC, 1 µM MinD, and 1 µM MinE), 100 mg/mL BSA, ADP and the ATP generation system consisted of 10 mM creatine phosphate (CrP) and 0.1 µM creatine kinase (CK) (top panel) are encapsulated in artificial cells instead of ATP. At the initial state, Min proteins localize homogeneously in cytosol of artificial cells because of the lack of ATP (shown in green). ATP synthesis generates Min waves in artificial cells (bottom). (B) Time-lapse images of dynamics of msfGFP-MinC in artificial cells. White arrows indicate the beginning and the end of approximately one cycle of Min wave propagation in three different artificial cells. Time is in min:s. (C) Scatter plots of Min wave band width (left) and velocity (right) against artificial cell diameter. (n =20). Band width and velocity were measured 10 minutes after the onset of Min wave propagation. Linear regression lines with zero intercept are shown.

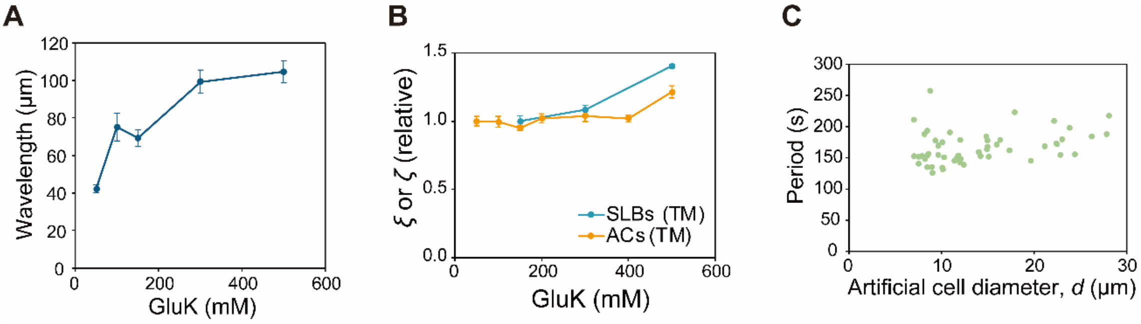

Fig. S6. Effects of K^+ concentration and spatial size on Min waves. (A) Wavelengths of Min waves on SLBs under varying GluK concentrations. Min waves were generated by adding 0.1 µM msfGFP-MinC, 1 µM MinD, 1 µM MinE, and 2.5 mM ATP to SLBs under different GluK concentrations. Data are presented as mean ± standard error (n = 10–17). (B) Minimal changes in ξ or ζ of Min wave at various GluK concentrations when the TM mutant is used. ξ on SLBs or ζ in artificial cells are analyzed in Min waves generated using 0.1 µM msfGFP-MinC, 1 µM MinD, 0.7 µM MinE$^{D45A/V49A/I74M}$ (TM mutant) with (for artificial cells and omitted for SLBs. Data are shown as mean ± standard error (SLBs: n = 22–28; artificial cells: n = 7–16). (C) Period of single Min waves in artificial cells of varying sizes. The period—the time required for a wave to travel around the cell—is plotted against artificial cell diameter (n = 53). Artificial cells were prepared by encapsulating 0.1 µM msfGFP-MinC, 1 µM MinD, 1 µM MinE, 2.5 mM ATP, and 100 mg/mL BSA are analyzed.

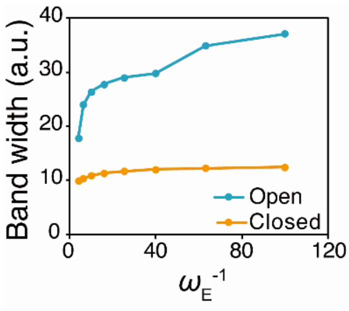

Fig. S7. Bandwidth of Min waves simulated by theoretical model under varying ω_E. Bandwidths of Min waves simulated using the Min wave model are plotted against ω_E^{-1}. While bandwidth varied with ω_E in the open system, it remained robust in the closed system.
